# Immature Platelet Fraction and Thrombin Generation: Preeclampsia Biomarkers

**DOI:** 10.1055/s-0042-1743100

**Published:** 2022-07-11

**Authors:** Daniela Moraes, Camila Milioni, Carolina Friske Vieira, Eveline Avila Parera, Bárbara Dewes Silva, Miriam Viviane Baron, Bartira Ercília Pinheiro da Costa, Carlos Eduardo Poli-de-Figueiredo

**Affiliations:** 1 Pontifícia Universidade Católica do Rio Grande do Sul, Porto Alegre, RS, Brazil

**Keywords:** platelets, platelet activation, hypertension pregnancy-induced, blood coagulation, complement system proteins, plaquetas, ativação plaquetária, hipertensão induzida pela gravidez, coagulação sanguínea, proteínas do sistema do complemento

## Abstract

Preeclampsia, a human pregnancy syndrome, is characterized by elevated blood pressure and proteinuria after the 20th week of gestation. Its etiology remains unknown, and its pathophysiological mechanisms are related to placental hypoperfusion, endothelial dysfunction, inflammation, and coagulation cascade activation. Recently, the role of the complement system has been considered. This syndrome is one of the main causes of maternal and fetal mortality and morbidity. This article discusses the hypothesis of preeclampsia being triggered by the occurrence of inadequate implantation of the syncytiotrophoblast, associated with bleeding during the first stage of pregnancy and with augmented thrombin generation. Thrombin activates platelets, increasing the release of antiangiogenic factors and activating the complement system, inducing the membrane attack complex (C5b9). Immature platelet fraction and thrombin generation may be possible blood biomarkers to help the early diagnosis of preeclampsia.

## Resumo

A pré-eclâmpsia, uma síndrome da gestação humana, é caracterizada por elevação da pressão arterial e proteinúria patológica após a 20ª semana de gestação. Sua etiologia permanece desconhecida, e seus mecanismos fisiopatológicos estão relacionados à hipoperfusão placentária, disfunção endotelial, inflamação, e ativação da cascata de coagulação. Recentemente, o papel do sistema do complemento foi considerado. Essa síndrome é uma das principais causas de morbidade e mortalidade materna e fetal. Este artigo discute a hipótese de a pré-eclâmpsia ser desencadeada pela ocorrência da implantação inadequada do sinciciotrofoblasto, associada ao sangramento durante o primeiro trimestre da gravidez com aumento da geração de trombina. A trombina ativa plaquetas, aumentando a liberação de fatores antiangiogênicos na circulação e ativando o sistema do complemento, especialmente o complexo de ataque de membrana (C5b9). Portanto, a fração de plaquetas imaturas e a geração de trombina podem ser possíveis biomarcadores sanguíneos para auxílio no diagnóstico precoce da pré-eclâmpsia.

## Introduction

Hypertensive disorders are very frequent complications in pregnancy. It is one of the main causes of maternal and fetal morbidity and mortality. 1 2 Preeclampsia (PE) is characterized by elevated blood pressure and pathological proteinuria after the 20th week of pregnancy. The incidence varies depending on where the study is being performed, but it is estimated to compromise from 2 to 8% of pregnancies. 1 3 4 The etiology is unknown, and its pathophysiological mechanisms are related to placental hypoperfusion, endothelial dysfunction, oxidative stress, inflammation, and coagulation changes. 5 6 7 8 9 10 11 12 13 14

## Theory

**Fig. 1:**
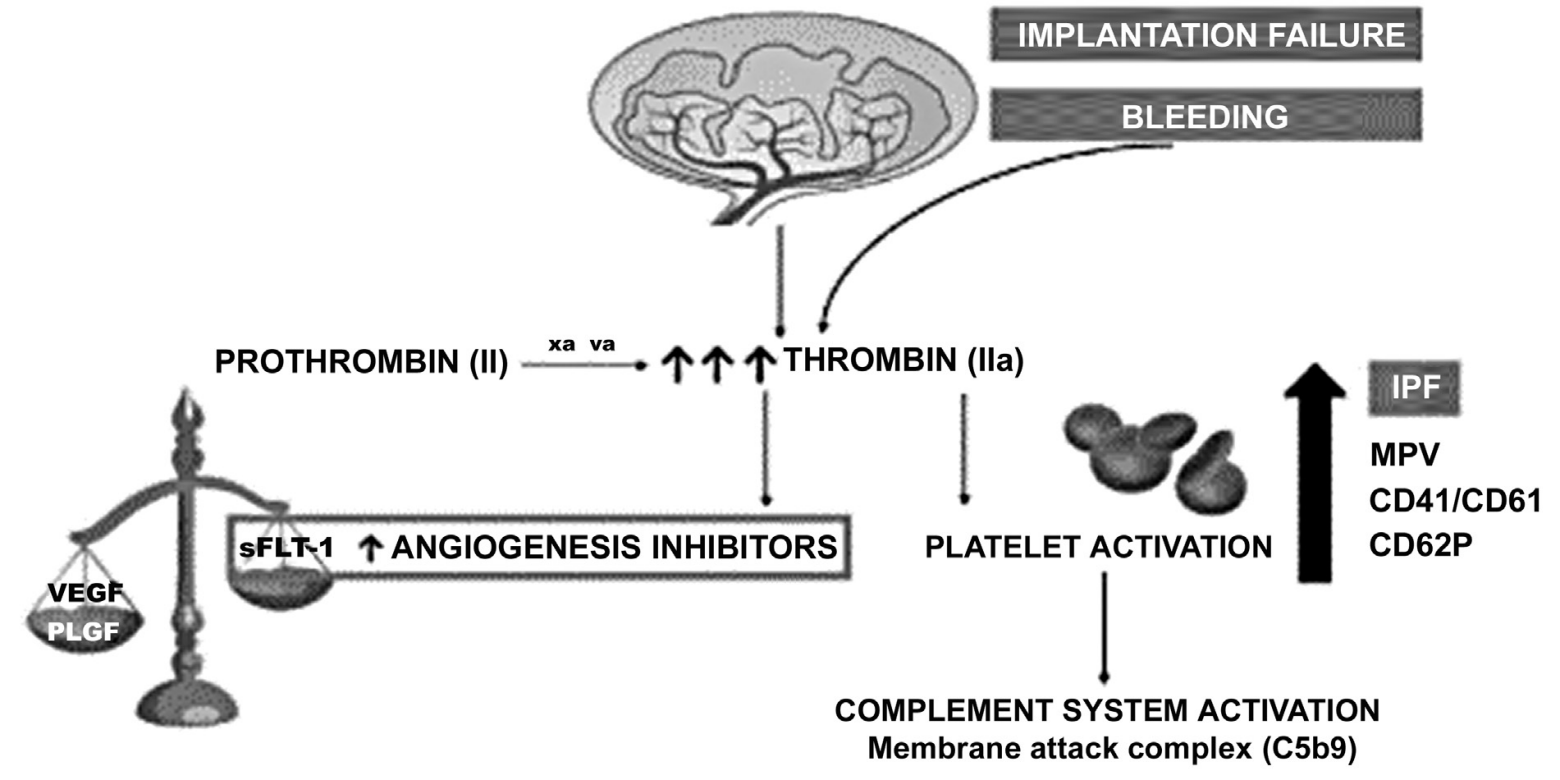
Scheme of the theory.

Defective implantation of the syncytiotrophoblast and bleeding in the first trimester of pregnancy contribute to increased thrombin generation, causing increased platelet activation and release of antiangiogenic factors in the maternal circulation (such as sFLT-1). The activation of platelets also triggers the complement system, membrane attack complex (C5b9) ( Fig. 1 ).

## Discussion

### Platelets and Preeclampsia

Hemostatic changes occur during pregnancy, shifting the balance in favor of hypercoagulability with an increased thrombosis risk. 15 These changes are aggravated in PE, as there is an abnormal activation of the hemostatic and immune system, which are responsible for most complications of the disease. In pregnant women with hypertensive disorder, especially PE, the total number of platelets and platelet parameters modifications, including the mean platelet volume (MPV) and the immature platelet fraction (IPF), platelet activation markers, and the complement system. Mean platelet volume, platelet distribution width (PDW), and IPF values are proportionally increased in relation to the severity of preeclampsia when compared with pregnant women without PE. 16 17 18 19 20 21 Thrombocytopenia results from increased platelet activation, aggregation, and consumption, and it maybe considered a platelet activation marker. 22 23

Platelets play an important role in the pathophysiology of PE, being responsible for coagulation and participating as an important inflammatory mediator. There is evidence of PE with platelet activation and increased platelet surface markers (CD62P) when comparing women with PE to healthy women. 24 25 26 In addition, there is an increase in CD41 expression in pregnant women with PE, evidencing platelet activation. 25

### Platelet and Thrombin Generation

Platelet activation may be due to increased thrombin generation. Thrombin is a multifunctional protease, responsible for coagulation cascade and one of the most potent platelet activators. Activation through thrombin generation causes degranulation and platelet activation, which displaces adhesion receptors to the cell surface and releases hemostatic and inflammatory mediators in the bloodstream, facilitating cell adhesion. 27 28 29 30 It is known that uterine bleeding or bruising at the moment of the syncytiotrophoblast implantation are associated with the development of PE and generates excess thrombin. 31 Bleeding in the first 20 weeks of pregnancy is a common complication, affecting about 1 in 5 pregnant women. 32 It has clinical relevance, as these patients develop an increased risk for unfavorable outcomes, mainly placental abruption, low birth weight, and premature birth. 33 34 As bleeding in pregnant women can be used as an early marker of placental dysfunction, there are studies associating bleeding with the development of PE. 25 However, findings remain conflicting. On the one hand, some authors disclosed a 35 to 40% increase in the risk of developing PE in patients with mild bleeding in the first stage of pregnancy when compared with pregnant women who did not present bleeding. 35 36 On the other hand, Smits et al. 31 found no association between bleeding (mild or severe) and the development of PE in primiparous women at low risk. However, among women with bleeding disorders, the results indicated that the analysis of intensity, pattern, and frequency of bleeding may indicate the risk of subsequent development of PE.

There are some studies showing association between thrombin generation increase and the pathogenesis of PE. 37 38 The excess of thrombin generated due to hemorrhage during placental development increases the expression of soluble feline McDonough sarcoma-like tyrosine kinase-1 (sFlt-1) by the trophoblast through the activation of the PAR-1/NADPH oxidase/ROS signaling pathway (specific receptors activated by proteinase). 39

There is evidence shown by the increased generation of thrombin in pregnant women with PE. 40 41 This activation induces neutrophil recruitment, activation, and oxidation. The excess of tissue factor binds to platelets, causing ADP release. This release increases thrombin generation, which has a high affinity for PAR-1 in the syncytiotrophoblast, platelets, and neutrophils, thus causing cell activation. 39

Thus, thrombin increases the secretion of sFlt-1. Soluble feline McDonough sarcoma-like tyrosine kinase-1is a receptor protein produced by syncytiotrophoblast, and its concentration in normal pregnancies is only a few times higher than that of placental growth factor (PlGF). It is related to the maternal endothelial dysfunction, a PE feature. 5 42 In hypoxia or inadequate perfusion of the placenta, the trophoblast produces a large quantity of sFlt-1, and its concentration in the maternal bloodstream is, at least, 12 times higher than the concentration of PlGF. 43

**Fig. 2:**
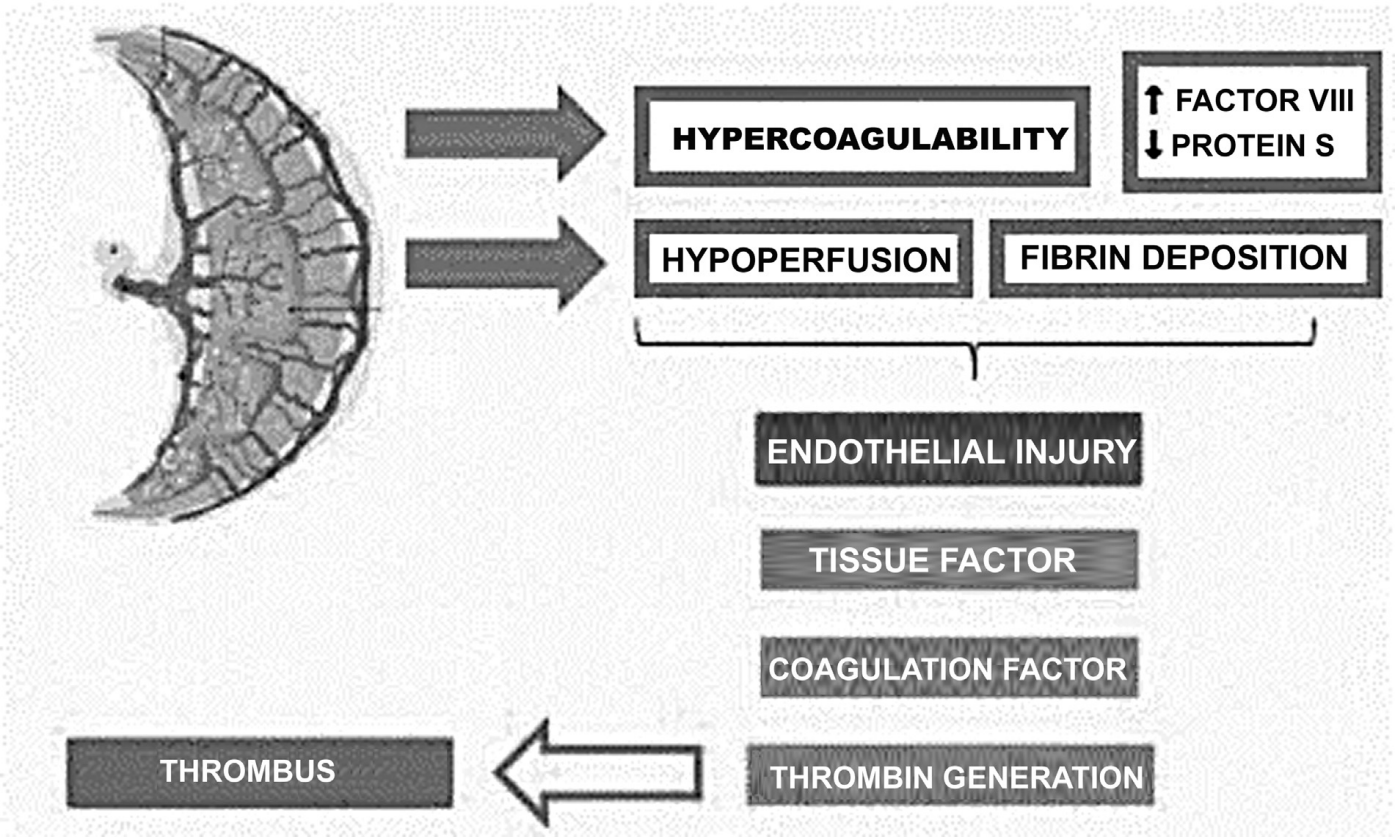
Factors involved in thrombus generation in preeclampsia.

Increased SFlt-1 in maternal circulation is one of the elements that determines the PE maternal multisystemic syndrome. These changes in sFlt-1 concentration precede the onset of clinical and laboratory symptoms in preeclamptic women by ∼ 5 to 6 weeks. 44 Therefore, laboratory tests to measure platelet activation and thrombin generation along with the sFlt-1 measurement could contribute to an early diagnosis of PE syndrome ( Fig. 2 ).

### Platelets and Complement System Activation

In addition, evidence brought by few studies shows the involvement of the excessive increase in tissue factor (TF) with the activation of proteins C3 and C5 of the complement system. This activation is probably due to the stress generated by the syncytiotrophoblast. Although TF is important for placental development, its increase during trophoblast implantation and tissue hemorrhage exarcebates the activation of coagulation cascade, which has been the first hypothesis of abnormal implantation of trophoblast. 45

**Fig. 3:**
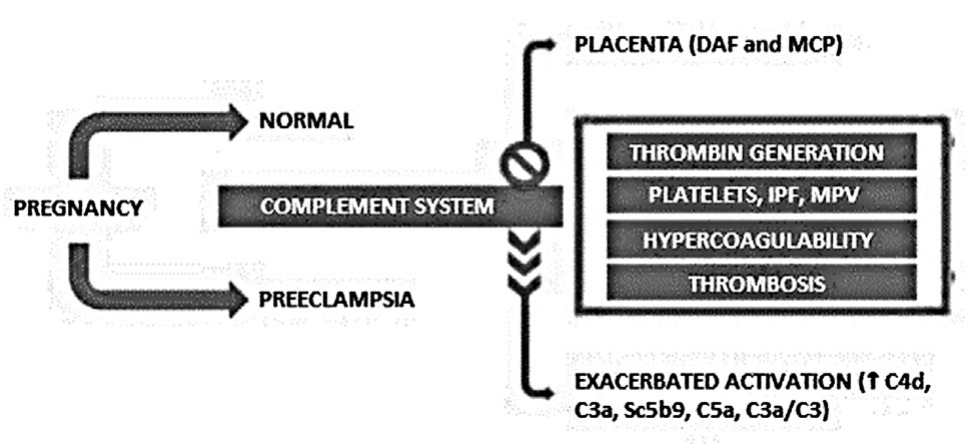
Complement cascade in normal pregnancy and preeclampsia.

The activated platelets trigger the alternative complement pathway, especially the membrane attack complex (C5b9). And the activation of complement proteins may help to trigger PE and hemolysis, elevated liver enzymes, low platelet count (HELLP) syndrome. Burwick et al. 46 showed an increase in plasma concentration of C5b9 complement proteins in patients with gestational hypertension. The activation of the membrane attack complex in hypertensive disorders reflects endothelial dysfunction and systemic inflammation ( Fig. 3 ).

## Conclusion

Platelets play a fundamental role in the pathophysiology of PE. We suggest that platelet activation in preeclamptic pregnancy is caused by the excess generation of thrombin associated with bleeding in the first trimester, increasing the release of antiangiogenic factors and activating the complement system before the onset of the clinical symptoms of the syndrome. Although platelet activation and increased IPF is confirmed in pregnant women who develop PE, these tests are not routinely performed for diagnosis. Laboratory essays for measuring IPF and thrombin generation are simple and easily accessible in laboratories that use advanced technology to perform them. They may be useful for the early diagnosis of this syndrome and the management of patients. Therefore, we believe further studies focused on these laboratory tests are required to enable early diagnosis and treatment of the disease.
